# Real-world effectiveness and safety of sofosbuvir/velpatasvir and ledipasvir/sofosbuvir hepatitis C treatment in a single centre in Germany

**DOI:** 10.1371/journal.pone.0214795

**Published:** 2019-04-04

**Authors:** Peter Buggisch, Karsten Wursthorn, Albrecht Stoehr, Petar K. Atanasov, Romain Supiot, Janet Lee, Jie Ting, Joerg Petersen

**Affiliations:** 1 Asklepios Klinik St. Georg Haus L, IFI Institut für Interdisziplinäre Medizin, Hamburg, Germany; 2 Amaris Consulting, London, United Kingdom; 3 Gilead Sciences, Inc., Foster City, CA, United States of America; National Taiwan University Hospital, TAIWAN

## Abstract

**Background:**

Newer direct-acting antiviral therapies are increasingly becoming the therapy of choice in patients with hepatitis C virus (HCV) infection. Here, we report the safety and effectiveness of sofosbuvir/velpatasvir (SOF/VEL) and ledipasvir/sofosbuvir (LDV/SOF) in real-world cohorts in Germany.

**Methods:**

Patients initiated on SOF/VEL 12 weeks or LDV/SOF 8, 12 or 24 weeks regimens in a single German centre were included in this study. Data on treatment outcomes and adverse events (AE) were analysed in patients with available sustained virologic response 12 weeks after cessation of treatment (SVR12) information overall and by subgroups.

**Results:**

This study included 115 patients who received SOF/VEL from July-2016 to July-2017, and 249 patients who received LDV/SOF from November-2014 to September-2015. Overall, SVR12 was achieved in 99% of patients on SOF/VEL ± ribavirin 12 weeks independent of HCV genotype, treatment history, or cirrhosis status, and in 96% of patients treated with LDV/SOF 8 weeks or LDV/SOF ± ribavirin 12 or 24 weeks. In genotype 1 treatment-naïve, non-cirrhotic patients, ≥99% achieved SVR12 across SOF/VEL and LDV/SOF regimens. Likewise, 100% of genotype 3-cirrhotic patients on SOF/VEL ± ribavirin regimens achieved SVR12. Grade 3/4 AE were reported in 13 (5.2%) patients on LDV/SOF and in 1 (<1%) patient on SOF/VEL.

**Conclusion:**

Overall, SOF/VEL and LDV/SOF achieved high SVR rates in a broad patient population. We showed the effectiveness of SOF/VEL as a pan-genotypic regimen, and regardless of treatment history or cirrhosis status. Use of such therapies improves outcomes and contributes towards the global efforts to eradicate HCV.

## Introduction

Hepatitis C virus (HCV) infection remains a public health issue with a global prevalence of 1%, amounting to approximately 71 million infected people worldwide [1]. In Germany, more than 245,000 people are estimated to be infected with the virus [2].

Approved in 2014, the efficacy and safety of ledipasvir/sofosbuvir (LDV/SOF) single tablet regimen (STR) in chronic hepatitis C (CHC) treatment have been demonstrated in a number of clinical trials, with ≥ 94% of patients achieving sustained virologic response 12 weeks after cessation of treatment (SVR12) across patient HCV genotypes (GT) 1,4,5, and 6, treatment history (treatment-naïve [TN] or treatment-experienced [TE]), or cirrhosis status [3–9]. The European Summary of Product Characteristics (SmPC) recommends LDV/SOF ± ribavirin (RBV) for adult patients with HCV GT 1, 3, 4, 5, and 6, with treatment duration of 8, 12, or 24 weeks, depending on HCV GT, treatment history, or cirrhosis status [10].

Sofosbuvir/velpatasvir (SOF/VEL), approved in 2016, is the first pan-genotypic, protease inhibitor (PI)-free, all-oral STR with a 12-week treatment duration across all CHC patients, independent of cirrhosis status, treatment history, or baseline resistance-associated substitutions (RAS) [11]. As such, it is one of the direct-acting antivirals (DAAs) recently recommended by World Health Organization (WHO) for the treatment and elimination of HCV [12]. In clinical trials, SOF/VEL demonstrated high SVR12 rates of 94–100% regardless of patient HCV GT, treatment history, cirrhosis status, or co-infection status [13–16]. Real-world patient cohorts treated with SOF/VEL regimens further corroborated these findings, reporting similarly high effectiveness and safety profiles across patient types as that observed in the clinical trials [17, 18].

Although several studies have confirmed the real-world effectiveness and safety of DAAs across different HCV infected subpopulations in Germany [19, 20], real-world outcomes of patients treated with SOF/VEL in a broad patient population are limited. To our knowledge, only one other study has been published on treatment with SOF/VEL among German patients, showing high SVR12 rates in HCV GT 3 (99.5% in the per protocol analysis) [21]. Our study reports the safety and effectiveness of SOF/VEL and LDV/SOF regimens in a real-world CHC patient population of mixed genotype, treatment history, and cirrhotic status treated at a single centre in Germany.

## Materials and methods

### Study design and patients

This was a retrospective observational single-centre study of consecutive patients treated for CHC at the IFI Institut für Interdisziplinäre Medizin, Hamburg, Germany, with either SOF/VEL or LDV/SOF, ± RBV. Patients who completed treatment and have valid SVR12 status, or discontinued treatment due to adverse events (AE) or lack of adherence were included in the study.

### Data collection

Baseline data were collected from patients’ case notes and included patient characteristics at initiation of SOF/VEL and LDV/SOF therapies (age, gender, ethnicity, GT, co-infection with HIV or HBV, baseline HCV RNA, blood test results, liver disease status, comorbidities), and previous HCV therapies (number and type of treatment, as well as associated response). Data related to treatment outcomes and AE were collected during patients’ follow up and assessed at data cut-off. HCV RNA was qualitatively measured by Roche COBAS AmpliPrep/COBAS TaqMan with a cut-off of <12 IU/ml. Fibrosis was measured by FibroScan with cut-off values for METAVIR stage F3 or less of ≤12.3kPa. RAS testing were performed after treatment with SOF/VEL or LDV/SOF, following previously documented methods [22, 23].

### Assessment of outcomes

The primary outcome of interest was the proportion of patients achieving SVR12. The incidence and type of grade 3 or 4 AEs, treatment discontinuation and/or hospitalisation were collected by the study centre through a standardised questionnaire and quality-controlled by a study coordinator. The relationship of HCV therapy to each AE was assessed by the study investigator and categorised as “probably”, “possibly”, “unrelated” or “unknown”. Non-adherence was assessed upon the discretion of the investigators and based on patient adherence to medical appointments, patient statements, and congruence to the prescriptions.

### Statistical analysis

Descriptive statistics were used to analyse the outcomes according to treatment duration (8 weeks, 12 weeks and 24 weeks), GT, treatment history (TN vs. TE), and METAVIR stage (F0, F1, F2, F3, and F4) at baseline. Significant differences at baseline between treatment arms for continuous variables were tested through an ANOVA procedure. Chi-squared test was performed for categorical variables when the number of patients per category was big enough. The proportion of patients achieving SVR12 was determined overall, well as by treatment duration, treatment history, and METAVIR stage.

## Results

### Baseline characteristics

The study included 115 patients on SOF/VEL and 249 patients on LDV/SOF with available SVR12 data at end of follow-up, or who had discontinued treatment due to AE or lack of adherence (Table 1). Median age of patients ranged from 50–56 years across different treatment regimens, and proportion of male patients ranged from 42.6% in LDV/SOF 8 weeks, to 85.7% in SOF/VEL 12 weeks + RBV. In terms of GT distribution, the majority of patients on SOF/VEL regimens were GT3 (73.9%), while most patients on LDV/SOF regimens were GT1 (85.9%).

**Table 1:** Baseline characteristics of chronic hepatitis C patients treated at a single centre in Germany.

Baseline characteristics	SOF/VEL 12 weeks (n = 80)	SOF/VEL+RBV 12 weeks (n = 35)	LDV/SOF 8 weeks (n = 129)	LDV/SOF±RBV 12 or 24 weeks (n = 120)
Age, Median (Min-Max) ┼	52 (26–76)	50 (28–68)	51 (22–77)	56 (28–79)
Males, n (%) ╪	50 (62.5)	30 (85.7)	55 (42.6)	78 (65.0)
Ethnic group, n (%)				
White	71 (88.8)	33 (94.3)	128 (99.2)	120 (100)
Other	9 (11.2)	2 (5.7)	1 (0.8)	0 (0.0)
Genotype, n (%)				
1	11 (13.7)	1 (2.9)	127 (98.5)	87 (72.5)
2	12 (15.0)	0 (0.0)	0 (0.0)	0 (0.0)
3	51 (63.8)	34 (97.1)	0 (0.0)	24 (20.0)
4	1 (1.2)	0 (0.0)	2 (1.5)	9 (7.5)
5	2 (2.5)	0 (0.0)	0 (0.0)	0 (0.0)
6	3 (3.8)	0 (0.0)	0 (0.0)	0 (0.0)
METAVIR Score, n (%) ╪				
F0	28 (35.0)	0 (0.0)	71 (55.0)	27 (22.5)
F1	10 (12.5)	0 (0.0)	28 (21.7)	11 (9.1)
F2	14 (17.5)	0 (0.0)	20 (15.5)	9 (7.5)
F3	11 (13.8)	2 (5.7)	10 (7.8)	17 (14.2)
F4	17 (21.2)	33 (94.3)	0 (0.0)	56 (46.7)
F4 and decompensated	1 (1.2)	6 (17.1)	0 (0.0)	3 (2.5)
Biological measures ┼				
Baseline HCV RNA (log10 IU/ml) Median (Q1-3; Min-Max)	6.07 (5.17–6.49; 1.81–7.56)	5.84 (4.96–6.55; 2.73–6.98)	5.87 (5.88–5.40; 1.04–7.27)	6.01 (5.44–6.37; 2.87–7.52)
Baseline Bilirubin (mg/dL) Median (Q1-3; Min-Max)	0.55 (0.40–0.75; 0.20–1.80)	0.7 (0.50–0.90; 0.30–2.90)	0.5 (0.40–0.70; 0.20–1.80)	0.6 (0.40–0.90; 0.20–4.10)
Baseline Albumin (g/L) Median (Q1-3; Min-Max)	38.85 (35.45–41.50; 26.00–50.10)	35.85 (32.5–38.70; 23.60–44.20)	39.2 (37.30–41.50; 0.00–55.70)	37.4 (34.00–39.60; 22.40–48.30)
Baseline Platelets (10^9^/L) Median (Q1-3; Min-Max)	205 (139.50–247.00; 34.00–444.00)	107 (67.00–145.00; 25.00–267.00)	224 (200.00–258.00; 95.00–426.00)	167 (126.00–241.00; 25.00–890.00)
Platelet count >150 x 10^9^/L, n (%)	57 (71.3)	8 (22.9)	120 (93.0)	72 (60.0)
Previous treatment status, n (%) ╪				
TN	70 (87.5)	26 (74.3)	126 (97.7)	44 (36.7)
TE	10 (12.5)	9 (25.7)	3 (2.3)	76 (63.3)
No previous DAA therapy	8 (80.0)	7 (77.8)	3 (100)	70 (92.1)
Previous DAA therapy	2 (20.0)	2 (22.2)	0 (0.0)	6 (7.9)
Previous NS5A	1 (50.0)	1 (50.0)	0 (0.0)	2 (33.3)
Previous non-NS5A	1 (50.0)	1 (50.0)	0 (0.0)	4 (66.7)
Presence of co-infection, n (%)				
HIV	6 (7.5)	0 (0.0)	5 (3.9)	7 (5.8)
HBV	0 (0.0)	0 (0.0)	0 (0.0)	3 (2.5)

DAA: direct-acting antiviral; dL: deciliter; g: gram; HBV: hepatitis B virus; HCV: hepatitis C virus; HIV: human immunodeficiency virus; IU: international unit; L: liter; LDV: ledipasvir; mg: milligram; ml: milliliter; NS5A: nonstructural protein 5A; Q1-3: first quartile–third quartile; RBV: ribavirin; RNA: ribonucleic acid; SOF: sofosbuvir; TE: treatment-experienced; TN: treatment-naive; VEL: velpatasvir

┼ Continuous variables: significant difference between treatment arms (at the significance level of 0.05) was found for the age, baseline bilirubin, baseline albumin and baseline platelets whereas there was no significant difference between treatment arms for baseline HCV RNA

╪ Categorical variables: significant difference between treatment arms (at significance level of 0.05) was found for the gender, the experienced status and the METAVIR score

The majority of patients treated with SOF/VEL or LDV/SOF 8 weeks were TN (range: 74.3% among SOF/VEL 12 weeks + RBV, to 97.7% among LDV/SOF 8 weeks), while most patients treated with LDV/SOF ± RBV 12 or 24 weeks were TE (63.3%). Among the TE patients across all regimens in this study, most did not receive a previous DAA (range: 77.8% among SOF/VEL 12 weeks + RBV, to 100% among LDV/SOF 8 weeks). Distribution of cirrhotic patients also differed by regimens, comprising the majority of patients treated with SOF/VEL 12 weeks + RBV (94.3%), followed by LDV/SOF ± RBV 12 or 24 weeks (46.7%), and SOF/VEL 12 weeks (21.2%).

Presence of HIV co-infection was reported in 5.2% (n = 6) of SOF/VEL 12 weeks patients, and 4.8% (n = 12) of LDV/SOF patients (5 on LDV/SOF 8 weeks, 7 on LDV/SOF ± RBV 12 or 24 weeks). Presence of HBV co-infection was reported in 1.2% (n = 3) of LDV/SOF patients (2 on LDV/SOF 8 weeks, 1 on LDV/SOF ± RBV 12 or 24 weeks).

### Effectiveness results

**Fig 1:**
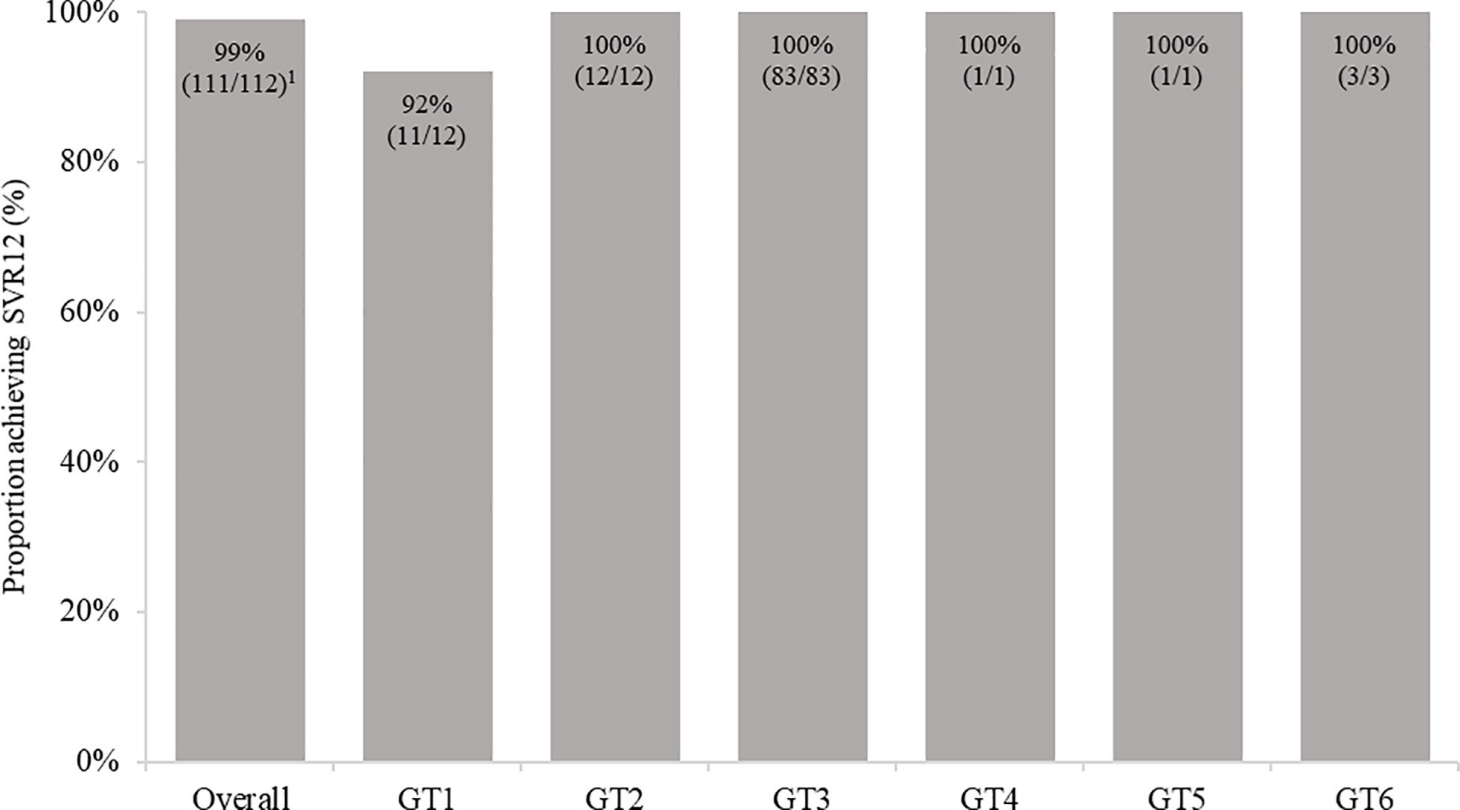
Proportion achieving SVR12 among SOF/VEL patients overall, and by genotype treated at a single centre in Germany. GT1: genotype 1; GT2: genotype 2; GT3: genotype 3; GT4: genotype 4; GT5: genotype 5; GT6: genotype 6; SVR12: sustained virologic response at 12 weeks. ^1^One patient did not achieve SVR with the following characteristics; male, 69 years old, white, GT1a, F4-CC and TN.

Among the 115 patients receiving SOF/VEL regimens, 80 patients were treated with SOF/VEL 12 weeks, and 35 with SOF/VEL + RBV 12 weeks. Three SOF/VEL patients discontinued treatment; 2 SOF/VEL without RBV patients (1 due to lack of adherence and 1 due to AEs [sleep disorder and mouth dryness]) and 1 SOF/VEL + RBV patient (due to lack of adherence). Thus, a total of 112 patients treated with SOF/VEL regimens completed the full treatment course and were included in the final per-protocol analyses (based on patients who completed treatment and had valid SVR12 data). SVR12 was achieved in 99.1% (111/112) of patients overall on SOF/VEL ± RBV 12 weeks, across all GT and patient subpopulations (Fig 1). The patient who did not achieve SVR12 was a 69 year-old who was GT1, cirrhotic, TN, treated without RBV, and had the following RAS identified: Q80 K, Y93H, Y561H. Similar findings were observed in an intention-to-treat analysis (based on patients who completed treatment and had valid SVR12 data, or discontinued treatment due to AE or lack of adherence), with SVR12 achieved in 96.5% (111/115) of SOF/VEL ± RBV 12 weeks patients.

Among the 249 LDV/SOF patients participating in the study, 129 (49.8%) received LDV/SOF 8 weeks and 120 (50.2%) received LDV/SOF ± RBV for 12 or 24 weeks. All LDV/SOF patients completed treatment and had valid SVR12 data, and were included in the final per-protocol analyses. Overall SVR12 rate was 99.2% (128/129) for patients on LDV/SOF 8 weeks, and 93% (111/120) for patients on LDV/SOF ± RBV 12 or 24 weeks. Among the 9 patients who did not achieve SVR12 under LDV/SOF ± RBV 12 or 24 weeks regimens, 6 were treated with LDV/SOF ± RBV 12 weeks (4 patients were GT 1, TE [3 cirrhotics, 1 non-cirrhotic]; 2 patients were GT 3 and cirrhotic [1 TE, 1 TN]), and the remaining 3 with LDV/SOF + RBV 24 weeks (1 patient was GT 1, cirrhotic, TE; 2 patients were GT 3 and cirrhotic [1 TE, 1 TN]). RAS mutations were observed in 7 out of these 9 patients, and one patient had missing RAS data: L31 M (n = 2), N558 S (n = 1), Q 30 R, H 58Y (n = 1), Q80 L, L 31 M (n = 1), and Q 80K, Q 30, H58Y (n = 1).

**Fig 2:**
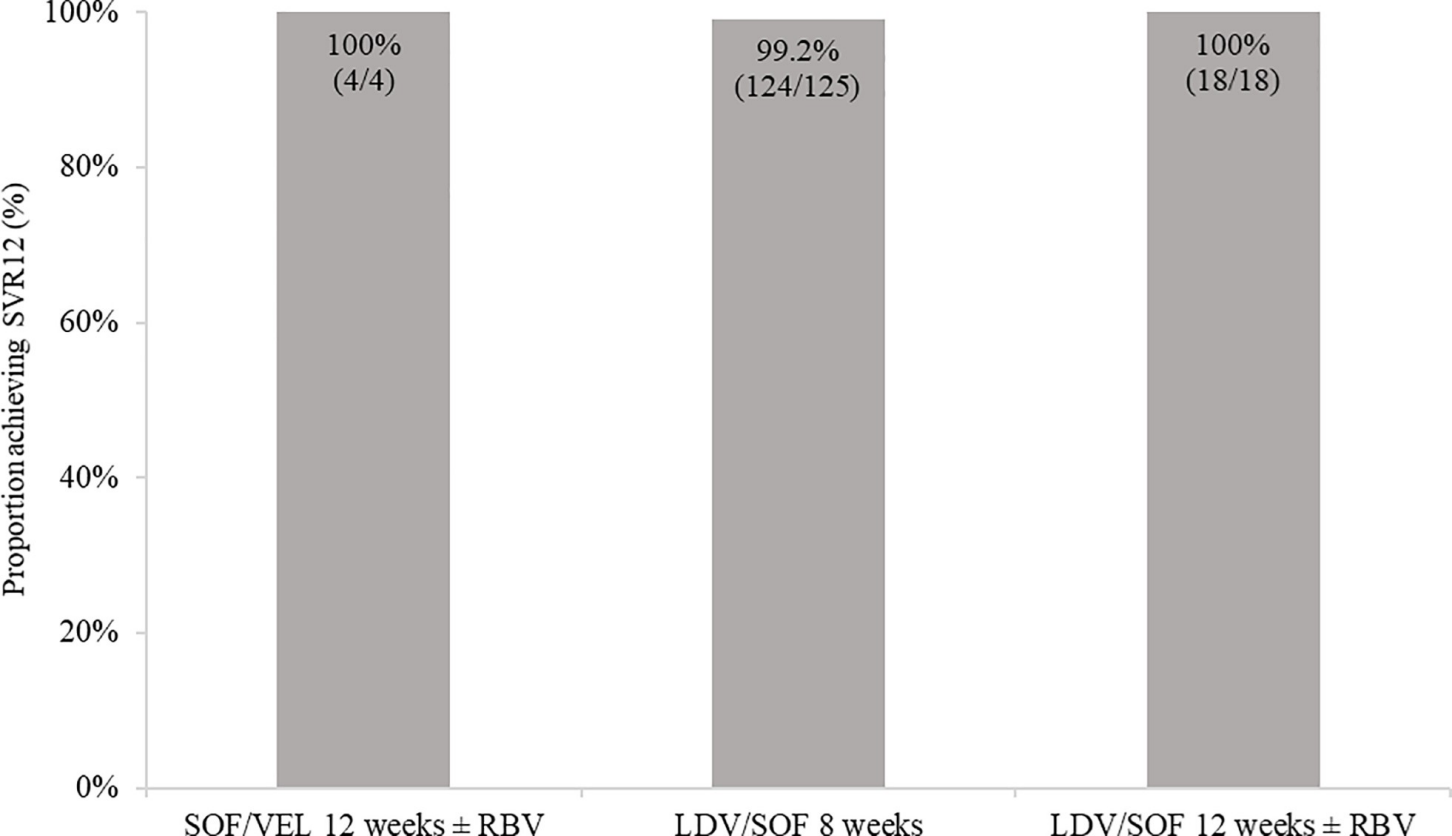
Proportion achieving SVR12 among GT1, treatment-naïve, non-cirrhotic patients on LDV/SOF or SOF/VEL treated at a single centre in Germany. LDV: ledipasvir; SOF: sofosbuvir; SVR12: sustained virological response at 12 weeks; VEL: velpatasvir.

Among GT1, TN, non-cirrhotic patients, >99% across all SOF/VEL or LDV/SOF regimens achieved SVR12 (Fig 2) Additionally, among the 40 cirrhotic GT 3 patients treated with SOF/VEL regimens (9 treated with SOF/VEL 12 weeks, 31 treated with SOF/VEL 12 weeks + RBV), all achieved SVR12.

Overall, 10 patients in this study previously failed a DAA therapy. Four of these patients were given SOF/VEL regimens (2 received a previous NS5A, and 2 received a previous non-NS5A), and 6 were given LDV/SOF 12 or 24 weeks ± RBV in this study (2 received a previous NS5A, and 4 received a previous non-NS5A). Among these patients, only 1 failed to achieve SVR12 (54 year old white woman on LDV/SOF 12 weeks + RBV, had GT1b and decompensated cirrhosis, was previously treated with daclatasvir, and had RAS testing after her first treatment with the following RAS identified: Q30R and L31M).

HIV co-infection was reported in 18 patients (6 on SOF/VEL regimens, and 12 on LDV/SOF regimens), and HBV co-infection in 3 LDV/SOF patients. One patient treated with LDV/SOF + RBV 12 weeks was co-infected with both HIV and HBV. All 20 co-infected patients achieved SVR12.

### Safety results

As shown in Table 2, only 1 (0.6%) SOF/VEL patient experienced any grade 3/4 AE, 13 (5.2%) LDV/SOF patients reported at least one grade 3/4 AE. In LDV/SOF patients, these AEs were ‘probably’ or ‘possibly’ related to treatment for 77% (10/13) of them and ‘probably’ or ‘possibly’ related to SOF-treatment for 23% of them (3/13). None of the AEs led to hospitalization or death.

**Table 2:** Adverse events reported among chronic hepatitis C patients treated at a single centre in Germany.

Adverse events, n (%)	SOF/VEL overall (n = 115)	LDV/SOF overall (n = 249)
Patients with any AEs (Grades 3 & 4)	1 (0.9)	13 (5.2)
AEs 'probably' or 'possibly' related to treatment	2/2 (100)	10/13 (76.9)
AEs 'possibly' or 'possibly' related to SOF-treatment	2/2 (100)	3/13 (23.1)
AEs leading to discontinuation	2/2 (100)	0/13 (0.0)
AEs leading to hospitalization	0/2 (0.0)	0/13 (0.0)
Death	0/2 (0.0)	0/13 (0.0)

AEs: adverse events; LDV: ledipasvir; SOF: sofosbuvir; VEL: velpatasvir

## Discussion

This study confirmed the high effectiveness of SOF/VEL and LDV/SOF therapies in the real world, with SVR12 results comparable to those achieved in clinical trials [3–8]. Compared with the availability of multiple real-world studies demonstrating effectiveness of LDV/SOF [24–27], fewer datasets have been available for SOF/VEL. We found high overall SVR12 rates in our real-world CHC population in Germany treated with SOF/VEL (99.1% achieved SVR12 across GT1-6) or LDV/SOF (96.0% achieved SVR12 across GT1, 3 and 4) regimens. SOF/VEL and LDV/SOF were equally effective in GT1, TN, non-cirrhotic patients, in whom SVR12 was >99.0% regardless of regimen. Additionally, SOF/VEL was also highly effective in GT3, cirrhotic patients, among whom 100% achieved SVR12 in our study.

Our finding of high overall SOF/VEL effectiveness across HCV GT, treatment history, or cirrhosis status is consistent with clinical and open-label trials results demonstrating high overall efficacy, with SVR12 ranging from 93% to 100% [16, 28]. They also support the findings of other real-world patient cohorts treated with SOF/VEL regimens, which reported high effectiveness and safety across patient types.

Although the SmPC recommends a 12-week SOF/VEL regimen for all patient population regardless of GT, treatment history, or cirrhosis status, only 10.7% (12/112) of patients treated with a SOF/VEL regimen were GT1. Moreover, patients who were GT1, TN, and non-cirrhotic comprised only 3.5% (4/112) of patients treated, all of whom achieved SVR12. This was possibly driven by recommendations in Germany leading to physicians selecting LDV/SOF 8 weeks for eligible GT1 patients, and SOF/VEL mainly for GT3 patients [29]. The WHO now recommends pan-genotypic regimens such as SOF/VEL as one of the safe and highly effective treatments for all adult CHC patients to achieve elimination of HCV infection [12]. Pan-genotypic DAAs can simplify the HCV care pathway by removing need for patient genotyping, and can reduce costs and loss to follow-up after diagnosis.

Our analysis also showed successful real-world use of SOF/VEL regimens in GT 3 cirrhotic patients, where 100% (40/40) of patients achieved SVR12, regardless of treatment history. Our finding is consistent with previous studies showing similarly high SVR 12 rates among GT 3 cirrhotic patients treated with SOF/VEL, from a retrospective pooled-analysis of the ASTRAL-1, -2, and -3 trials (91% achieved SVR12) [18], as well as from a German multicentre cohort study (98% achieved SVR12) [21].

There are a number of limitations associated with our study inherent to real-world, uncontrolled, observational studies. First, patients included in this study were recruited from a single centre only, thus potentially affecting generalisability of results to other areas in Germany as practices and patients characteristics may differ. For instance, although LDV/SOF regimens were recommended by the SmPC for GT 1, 3, 4, 5, or 6 patients, depending on patient characteristics, our study only included patients with GT 1, 3, and 4. Nevertheless, for SOF/VEL regimens, we were able to demonstrate high real-world effectiveness across patient types, regardless of GT, treatment history, or cirrhosis status. Secondly, some patient subgroups are limited by small sample size (e.g regimens in combination with RBV), and thus should be interpreted with caution. Lastly, this study was not designed to assess the long-term clinical outcomes of patients who achieved SVR. However, other recent datasets have shown reductions in mortality, HCC, hepatic decompensation, and need for liver transplant, which has attendant positive health economic outcomes for single-payer health care systems such as Germany.

## Conclusion

Our study shows that consistent with clinical trial findings, SOF/VEL and LDV/SOF achieve high SVR in a diverse patient population. SOF/VEL as a 12 week pan-genotypic treatment option proved to be a potent and convenient option across a broad patient population. Previously known negative predictive factors did not impact the real-world effectiveness of this regimen in this study.

## Supporting information

S1 Table Proportion achieving SVR12 among SOF/VEL patients overall, and by genotype, treatment history, and cirrhosis status treated at a single centre in Germany. (DOCX)

S2 Table Listing of the adverse events reported among chronic hepatitis C patients treated at a single centre in Germany. AEs: adverse events; LDV: ledipasvir; SOF: sofosbuvir; VEL: velpatasvir.
(DOCX)
