# Supplementary material for: Real-world effectiveness and safety of sofosbuvir/velpatasvir and ledipasvir/sofosbuvir hepatitis C treatment in a single centre in Germany
Source: PLoS One. 2019 Apr 4;14(4):e0214795. doi: 10.1371/journal.pone.0214795 (PMC6448908; doi:10.1371/journal.pone.0214795)
Supplement: S1 Table — (DOCX) [file pone.0214795.s001.docx]

**S1 Table. Proportion achieving SVR12 among SOF/VEL patients overall, and by genotype, treatment history, and cirrhosis status treated at a single centre in Germany**

| **Genotype** | **SOF/VEL 12 weeks  (n=78)** | **SOF/VEL + RBV 12 weeks  (n=34)** |
| --- | --- | --- |
| **Overall** | | |
| Overall, % SVR (n/N) | 98.7% (77/78) | 100% (34/34) |
| Naïve, % SVR (n/N) | 98.5% (67/68) | 100% (25/25) |
| Experienced, % SVR (n/N) | 100% (10/10) | 100% (9/9) |
| Non-cirrhosis, % SVR (n/N) | 100% (61/61) | 100% (2/2) |
| Cirrhosis, % SVR (n/N) | 94.1% (16/17) | 100% (32/32) |
| **Genotype 1** | | |
| GT1 overall, % SVR (n/N) | 90.9% (10/11) | 100% (1/1) |
| Naïve, % SVR (n/N) | 88.9% (8/9) | 100% (1/1) |
| Experienced, % SVR (n/N) | 100% (2/2) | - |
| Non-cirrhosis, % SVR (n/N) | 100% (5/5) | - |
| Cirrhosis, % SVR (n/N) | 83.3% (5/6) | 100% (1/1) |
| **Genotype 2** | | |
| GT2 overall, % SVR (n/N) | 100% (12/12) | - |
| Naïve, % SVR (n/N) | 100% (11/11) | - |
| Experienced, % SVR (n/N) | 100% (1/1) | - |
| Non-cirrhosis, % SVR (n/N) | 100% (10/10) | - |
| Cirrhosis, % SVR (n/N) | 100% (2/2) | - |
| **Genotype 3** | | |
| GT3 overall, % SVR (n/N) | 100% (50/50) | 100% (33/33) |
| Naïve, % SVR (n/N) | 100% (44/44) | 100% (24/24) |
| Experienced, % SVR (n/N) | 100% (6/6) | 100% (9/9) |
| Non-cirrhosis, % SVR (n/N) | 100% (41/41) | 100% (2/2) |
| Cirrhosis, % SVR (n/N) | 100% (9/9) | 100% (31/31) |
| **Genotype 4** | | |
| GT4 overall, % SVR (n/N) | 100% (1/1) | - |
| **Genotype 5** | | |
| GT5 overall, % SVR (n/N) | 100% (1/1) | - |
| **Genotype 6** | | |
| GT6 overall, % SVR (n/N) | 100% (3/3) | - |
