# Supplementary material for: Real-world effectiveness and safety of sofosbuvir/velpatasvir and ledipasvir/sofosbuvir hepatitis C treatment in a single centre in Germany
Source: PLoS One. 2019 Apr 4;14(4):e0214795. doi: 10.1371/journal.pone.0214795 (PMC6448908; doi:10.1371/journal.pone.0214795)
Supplement: S2 Table — AEs: adverse events; LDV: ledipasvir; SOF: sofosbuvir; VEL: velpatasvir. (DOCX) [file pone.0214795.s002.docx]

**S2 Table. Listing of the adverse events reported among chronic hepatitis C patients treated at a single centre in Germany**

| **Adverse events** | **SOF/VEL  12 weeks overall (n=2)** | **LDV/SOF  overall (n=13)** |
| --- | --- | --- |
| AEs 'probably' or 'possibly' related to treatment | 2/2 (100) | 10/13 (76.9) |
| Anaemia | - | 3 |
| Flu | - | 1 |
| Dyspnoea | - | 1 |
| Irritation | - | 1 |
| Eruption | - | 1 |
| Cephalgia | - | 1 |
| Acute bronchitis | - | 1 |
| Headache | - | 1 |
| Nausea | - | 1 |
| Acute bronchitis with dyspnoea | - | 1 |
| Fatigue | - | 1 |
| Sleep disorder | 1 | - |
| Mouth dryness | 1 | - |
| AEs 'possibly' or 'possibly' related to SOF-treatment | 2/2 (100) | 3/13 (23.1) |
| Cephalgia | - | 1 |
| Headache | - | 1 |
| Nausea | - | 1 |
| Sleep disorder | 1 | - |
| Mouth dryness | 1 | - |
| AEs leading to discontinuation | 2/2 (100) | 0/13 (0.0) |
| Sleep disorder | 1 | - |
| Mouth dryness | 1 | - |
